# Fabrication and evaluation of porous and conductive nanofibrous scaffolds for nerve tissue engineering

**DOI:** 10.1007/s10856-021-06519-5

**Published:** 2021-04-13

**Authors:** Yasaman Pooshidani, Nastaran Zoghi, Mina Rajabi, Masoumeh Haghbin Nazarpak, Zahra Hassannejad

**Affiliations:** 1 Departmant of Biomedical Engineering, Amirkabir University of Technology (Tehran Polytechnic), Tehran, Iran; 2 Department of Biochemistry, Faculty of Biological Sciences, Tarbiat Modares University, Tehran, Iran; 3 Centre for Bioengineering and Nanomedicine, University of Otago, Dunedin, New Zealand; 4 New Technologies Research Center (NTRC), Amirkabir University of Technology (Tehran Polytechnic), Tehran, Iran; 5 Pediatric Urology and Regenerative Medicine Research Center, Tehran University of Medical Sciences, Tehran, Iran; 6 Sina Trauma and Surgery Research Center, Tehran University of Medical Sciences, Tehran, Iran

## Abstract

Peripheral nerve repair is still one of the major clinical challenges which has received a great deal of attention. Nerve tissue engineering is a novel treatment approach that provides a permissive environment for neural cells to overcome the constraints of repair. Conductivity and interconnected porosity are two required characteristics for a scaffold to be effective in nerve regeneration. In this study, we aimed to fabricate a conductive scaffold with controlled porosity using polycaprolactone (PCL) and chitosan (Chit), FDA approved materials for the use in implantable medical devices. A novel method of using tetrakis (hydroxymethyl) phosphonium chloride (THPC) and formaldehyde was applied for in situ synthesis of gold nanoparticles (AuNPs) on the scaffolds. In order to achieve desirable porosity, different percentage of polyethylene oxide (PEO) was used as sacrificial fiber. Fourier transform infrared spectroscopy (FTIR) and field emission scanning electron microscopy (FE-SEM) results demonstrated the complete removing of PEO from the scaffolds after washing and construction of interconnected porosities, respectively. Elemental and electrical analysis revealed the successful synthesis of AuNPs with uniform distribution and small average diameter on the PCL/Chit scaffold. Contact angle measurements showed the effect of porosity on hydrophilic properties of the scaffolds, where the porosity of 75–80% remarkably improved surface hydrophilicity. Finally, the effect of conductive nanofibrous scaffold on Schwann cells morphology and vaibility was investigated using FE-SEM and MTT assay, respectively. The results showed that these conductive scaffolds had no cytotoxic effect and support the spindle-shaped morphology of cells with elongated process which are typical of Schwann cell cultures.

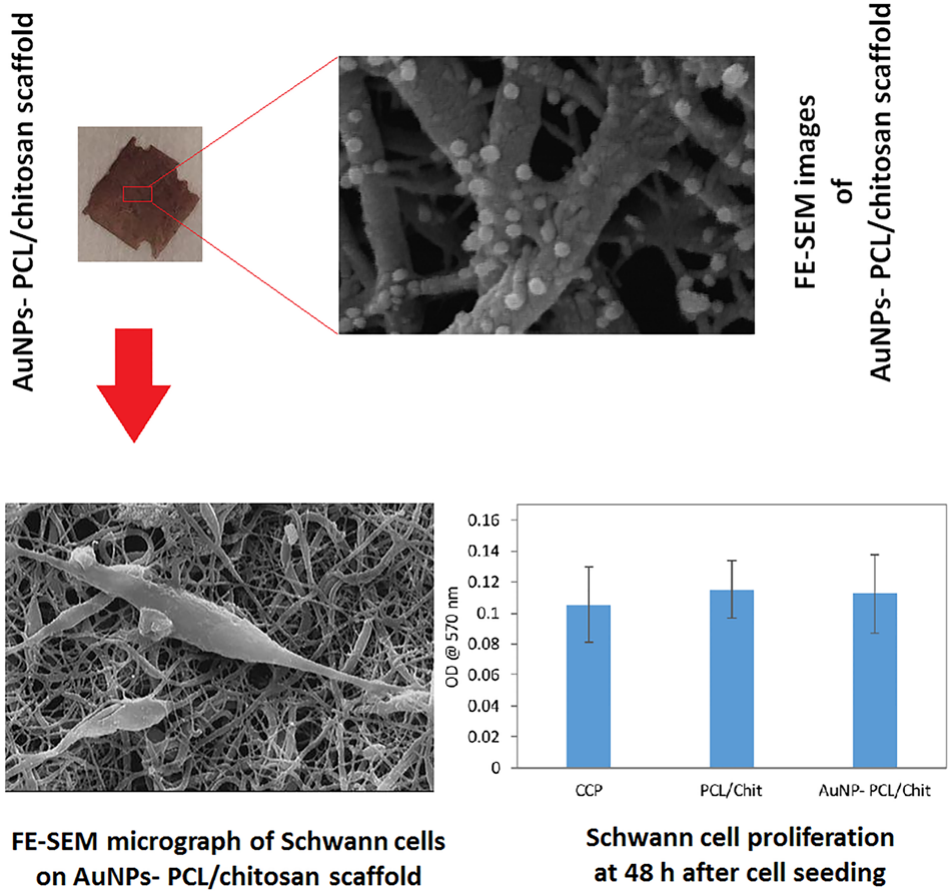

## Introduction

Peripheral nerve injury causes long-term disabilities for patients and also major socio-economic costs. The common approach to repair a short-distance gap in peripheral nerve is direct suturing of two stumps. In cases of long nerve gaps, implantation of autologous nerve grafts such as sural nerve to bridge the gap is still the gold standard, but it suffers from limited length, lack of donor nerves, morbidity of donor site and scar tissue invasion. The use of allogenic and xenogeneic tissues has also been suggested which eliminate the need of secondary surgeries on patients; however, they may arise the risk of disease transmission and immunogenicity problems. Nerve tissue engineering can be an alternative approach which provide a suitable and permissive microenvironment at the injury site using biocompatible scaffolds. An effective scaffold should provide required mechanical support for growing neurites, reduce scar tissue formation, and also chemical (e.g., release of nerve growth factors) and physical (e.g., topographical and electrical) signals [1–3].

The other important factor which potentially influences cell behavior and consequently tissue regeneration is scaffold porosity and pore size. Interconnected porous networks are essential for cell viability, proliferation and migration, nutrient exchange, vascularization and formation of new tissues [4]. Electrospinning is a well-known technique for generating nanometer- to micrometer-sized fibers with different orientations to mimic tissues extracellular matrix [5–7]. However, the main limitation of electrospinning method is the low porosity of the electrospun fibrous scaffolds. Incorporation of water soluble fibers in scaffolds during electrospinning process which could be subsequently washed out (i.e., sacrificial fibers) is a simple and controllable method for tuning the scaffold porosity [8]. Ifkovits et al. [9] used poly (ethylene oxide) (PEO) as the sacrificial polymer during electrospinning of poly (glycerol sebacate) and observed an increase in the scaffold porosity while maintaining the scaffold anisotropy which resulted fast integration of the scaffold with surrounding tissue compared to scaffolds prepared using conventional electrospinning method with lower porosity. Backer et al. [10] also showed that increasing the initial fraction of PEO, as sacrificial fibers, to the PCL nanofibrous scaffolds can enhance human meniscus fibrochondrocytes infiltration and improve cell distribution within the scaffolds.

The fact that the presence of bioelectricity plays an essential role in signal transduction in the nerve tissue has attracted great interest for fabrication of conductive substrates for improving cell growth and nerve tissue repair [11, 12]. Several studies have reported that electrical stimulation and conductive substrates can affect the morphology and function of neural cells [2, 13]. Our recent study also proved that gold nanoparticles (AuNPs) decorated PCL/Chit nanofibrous scaffold has proper electrical conductivity to increase the adhesion and growth of Schwann cells [14].

Schwann cells are peripheral glial cells which play an important role in nerve regeneration by excreting chemical cues to guide axon regrowth across the lesion. The biophysical and biochemical mechanisms of cell responses to electrical stimulation are very complex and almost unknown. However, there are supporting evidences showing electrical recognition by cells and signal transduction in which several cell membrane proteins not only sense but also use external electric filed to regulate the cellular functions [15, 16]. Electrical stimulation can accelerate Schwann cells proliferation, protein secretion, axon outgrowth along the injury site and consequently improve muscle innervation, therefore could be an effective approach to enhance nerve regeneration [17].

In the current study, we aimed to fabricate a nanofibrous PCL/Chit scaffold with interconnected porosity and electrical conductivity for nerve regeneration applications. For this purpose, scaffolds with different percentage of porosity were prepared using a triple-nozzle electrospinning device and sacrificial fiber method. Moreover, in order to increase conductivity, a novel method of in situ gold reduction by two reducing agents of tetrakis (hydroxymethyl) phosphonium chloride (THPC) and formaldehyde was used. Afterward, Schwann cells morphology and viability were evaluated to confirm the potential of using these PCL/chit nanofibrous scaffolds for nerve tissue engineering applications.

## Materials and methods

### Materials

Polycaprolactone (PCL, *M*_W_ = 80,000), sodium hydroxide (NaOH), potassium carbonate (K_2_CO_3_), formaldehyde (37%), acetic acid (99.8 wt%), formic acid (98–100 wt%), ethanol (99.6%), and ammonia (25%) were purchased from Merck. Chitosan (*M*_W_ = 200–300 kDa, acetylation degree of 15%), PEO (*M*_W_ = 200 kDa), tetrachloroauric acid trihydrate (HAuCl_4_·3H_2_O, ≥49% Au basis) and THPC (80%) were purchased from Sigma-Aldrich. The cell culture medium (DMEM/F12), fetal bovine serum (FBS), Trypsin, collagenase and bovine pituitary extract (BPE) were purchased from Gibco^TM^. All the materials were used as received and all reactions and solution preparation were carried out under ambient conditions unless otherwise stated.

### Preparation of PCL/Chit and PEO solutions

The PCL (10% wt/v) and chitosan (1% wt/v) were dissolved in a solvent mixture of acetic acid/formic acid (70:30 v/v%) and stirred with a medium speed for 3 h [14]. PEO was dissolved in ethanol and stirred for 6 h [18]. Finally, the electrospinning was conducted using different percentages of PEO solution (0, 20, 40, and 60%) to PCL/Chit solution.

### Fabrication of electrospun PCL/Chit and PEO nanofibers

An advanced triple-jet electrospinning device (model NF.Co.N/VI, Iran) was used to obtain different ratios of PEO and PCL/Chit in the electrospun fibrous composites. For all samples, distance from needle tip to collector and the flow rate were adjusted to 10 cm and 0.2 ml/h, respectively. The needle length and inner diameter were 5 cm and 1.19 mm, respectively. Applied voltage for the nozzle containing PCL/Chit solution was 18 kV and it was 13 kV for the nozzle containing PEO solution. The speed rotation of collector was set at 1250 rpm and linear speed of 4 mm/s [10, 18]. The electrospinning nozzles used for different PEO percentages are summarized in Table 1.

**Table 1:** Nozzles used for different PEO percentages

Sample name	PEO (%)	Nozzle 1	Nozzle 2	Nozzle 3
S-0% PEO	0	PCL/chitosan	PCL/chitosan	–
S-20% PEO	20	PCL/chitosan	PCL/chitosan	PEO
S-40 % PEO	40	PCL/chitosan	PEO	–
S-60% PEO	60	PCL/chitosan	PEO	PEO

### Stabilizing of nanofibrous scaffolds

In order to prevent the immediate dissolution of chitosan in aqueous medium, electrospun nanofiber mats were placed in an ammonium hydroxide solution (13%) for 15 min [19] which also led to detachment of fibrous mat from the lower aluminum foil. The electrospun nanofiber mats were then rinsed with deionized water until the pH of the rinsing water reached to 7. Subsequently, the samples were dried, weighed and kept in a desiccator [14, 20].

### Sacrificial fiber removal

In order to remove the PEO sacrificial fibers, samples with a determined weight (*M*_1_) were immersed in an aqueous solution of ethanol (70%) for 3 h and then were placed in deionized water for 1 h. Subsequently, scaffolds were dried in a desiccator for 24 h and re-weighed (*M*_2_) to determine the percentage of PEO removal using Eq. (1) [21]. The percentage was calculated using the samples which were fabricated in three independent experiments.

1 $${\mathrm{PEO}}({\mathrm{\% }}) = \frac{{{\mathrm{M}}1 - {\mathrm{M}}2}}{{{\mathrm{M}}1}} \times 100$$

### Preparation of AuNPs-decorated electrospun nanofibrous scaffolds

Synthesis of AuNPs on the PCL/Chit nanofibrous mats was conducted through a multistep in-situ reduction procedure. This procedure includes immobilization of the AuCl_4_^−^ ions on the PCL/Chit nanofibers using amino groups of chitosan and subsequently nucleation and growth of AuNPs using two reducing agents of THPC and formaldehyde [22]. Briefly, the PCL/Chit nanofiber mats were immersed in 2 ml gold aqueous solution (1% wt of HAuCl_4_·3H_2_O) and incubated at room temperature in the dark for 24 h to allow the electrostatic attachment of AuCl_4_^−^ ions to the amino groups of chitosan. Thereafter, the aqueous gold solution containing the electrospun mats was suddenly added to a reduction bath consists of 45 ml of deionized water, 0.5 ml of NaOH (1 M) aqueous solution and 1 ml of diluted THPC (12 μl of THPC 80% in 1 ml of deionized water). The samples were vigorously stirred for 5 min while the color immediately changed to purple. To prevent bending of the nanofiber mats, they were placed in a tissue basket and finally were washed three times with deionized water.

**Fig. 1:**
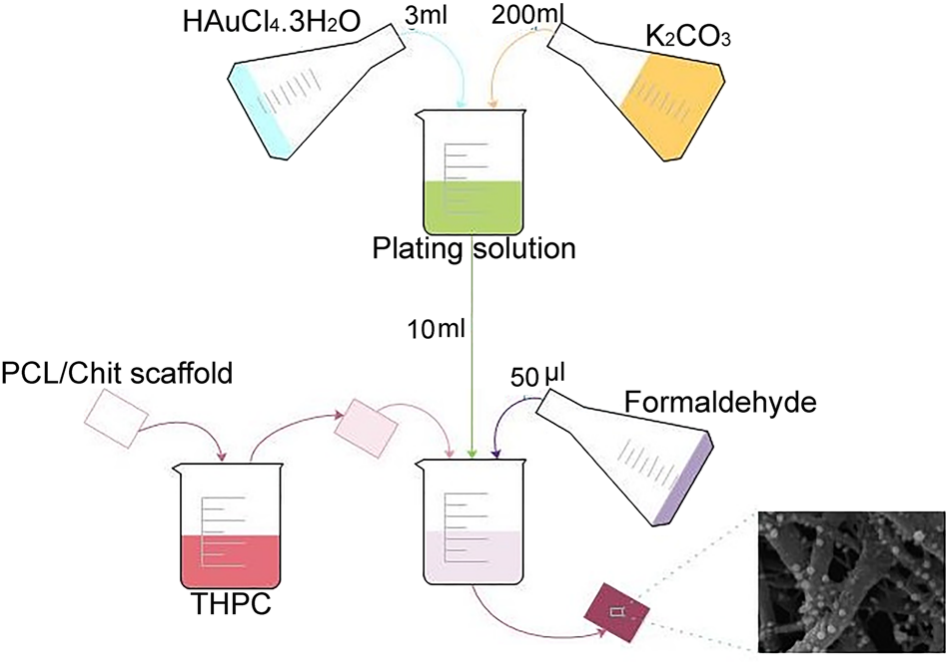
Schematic representation of the in-situ reduction of AuNPs

Further growth of AuNPs on the nanofibrous mats was carried out using the formaldehyde. For this purpose, a plating solution was prepared by mixing 3 ml AuCl_4_^−^ (1% wt/v) with 200 ml aqueous solution of K_2_CO_3_ (1.8 mM). The solution was stored at 4 °C for at least 24 h prior to use. Then, the samples obtained from the first step (i.e., using THPC as the reducing agent) were immersed in 10 ml of plating solution followed by addition of 50 µl formaldehyde 37%. Further reduction of gold nanoparticles was continued for 15 min which was visible through the color change of the solution from purple to brown. Extra formaldehyde was removed through three times washing with deionized water [22]. The process of in-situ AuNPs reduction is illustrated in Fig. 1.

### Field emission scanning electron microscopy (FE-SEM) and energy-dispersive X-ray analysis (EDX)

In order to evaluate the morphology, porosity, fiber diameter, size and size distribution of AuNPs; scaffolds were assessed by using an AIS 2100 FE-SEM (Hitachi S4160). The average fiber diameter was measured using NIH ImageJ software over randomly selected 100 fibers. Energy-dispersive X-ray analysis of the scaffolds was carried out for identification and quantification of the elemental composition (Ametek Element). Also, electron microscopy of Schwann cells seeded on the scaffold was conducted after fixation of cells using 4% paraformaldehyde and serial dehydration using ethanol.

### Porosity measurement

The porosity percentage was calculate using the samples prepared from three independent experiments and by two methods as explained in more detail below.

#### Liquid displacement method

In this method, scaffolds with determined weight were placed in a liquid that did not dissolve or swell the nanofibers. Ethanol is a nonpolar liquid which does not interfere with the polymeric fibers, hence it easily penetrates into the mat and occupies all the pores of the sample, and gives the total volume of pores. Weight of the nanofiber mats was measured before and after immersion in ethanol. The percentage of porosity was determined using the following equation:

2 $$P({\mathrm{\% }}) = \frac{{\left( {m_a - m_p} \right)/\rho _a}}{{\left( {m_a - m_p} \right)/\rho _a + m_p/\rho _p}} \times 100$$

where *m*_a_, *m*_p_, *ρ*_a_ and *ρ*_p_ are the mass of saturated mat, the mass of dried mat, the density of liquid (ethanol) and the density of polymer, respectively [23].

#### Apparent porosity

The apparent porosity (*P*_a_) of the scaffolds was calculated based on Eqs. (3) and (4), where *ρ*_a_ and *ρ*_b_ are apparent density and the bulk density of the scaffold, respectively, *m* represents the mass of scaffolds and *V* is the volume of the samples.

3 $$P_a\left( {\mathrm{\% }} \right) = \left[ {1 - \left( {\frac{{\rho _a}}{{\rho _b}}} \right)} \right] \times 100$$

4 $$\rho _a\left( {g/cm^3} \right) = \frac{m}{V}$$

The mean porosity size was obtained based on following formulas in which *L*, *D* and m are the length, average diameter and mass of the fiber, respectively [1].

5 $$L = \frac{m}{{\pi \rho _b\left( {\frac{D}{2}} \right)^2}}$$

6 $${\mathrm{log}}\left( {{\mathrm{MPS}}} \right) = - 0.37 \times {\mathrm{log}}\left( {\frac{{\mathrm{L}}}{{1000}}} \right) + 1.97$$

### Fourier transform infrared spectroscopy (FTIR)

The FTIR spectroscopy was carried out using a Nicolet device (Thermo Nivolet Corp., USA) to investigate the chemical composition of polymers, reactions, and the extent of sacrificial polymer (i.e., PEO) before and after washing.

### Electrical conductivity measurement

The DC electrical conductivity of the AuNP-decorated nanofibrous scaffolds was measured using a resistance meter (2602-A, Keithley) by the two-probe method under ambient condition. Three pieces of each scaffold were cut in the size of 1 × 10 mm^2^. The thickness of each specimen was measured by a micrometer with a precision of ±10 μm. Two sheets of copper with the thickness of 200 μm and dimension of 1 × 1 mm^2^ were placed on two sides of each scaffold and different electrical flow-voltage curves were obtained using the voltage range of 0–10 V. This measurement was performed using the samples which were obtained from two independent experiments in triplicate.

### Ultraviolet and visible (UV–Vis) spectroscopy

The optical properties of the AuNP-decorated scaffolds were determined by using a UV–Vis spectrophotometer (AvaSpec-UV/VIS/NIR, Netherlands) at room temperature, using the wavelength range between 520 and 600 nm to identify the resonance peaks of in situ synthesized AuNPs using THPC alone or in combination with formaldehyde.

### Water contact angle (WCA) measurement

Hydrophilicity of the PCL/Chit electrospun scaffolds which were prepared using different percentages of PEO (i.e., 0, 20, 40, and 60%) was determined based on sessile drop method using a G10 contact goniometer (Kruss, Germany) by dispensing 5 μl drops of deionized water on the samples surfaces at room temperature. The WCA values were reported as average of four measurements.

### Schwann cell isolation, seeding, and cultivation

Schwann cells were extracted from sciatic nerves of the 2- to 3- day-old Wistar rats and then were purified according to our previous protocol [1]. Briefly, sciatic nerves were extracted, the epineurium was removed, and then nerves were split into several explants and placed in PDL-coated plates. The culture medium of DMEM/F12 supplemented with FBS (10%), penicillin/streptomycin and 20 μg/mL of BPE was added to the plates and then the plates were incubated in a humidified 5% CO_2_ chamber at 37 °C. The explants were sub-cultured two to three times during 2 weeks to reduce the possibility of contamination with fibroblasts. Afterward, the explants were digested with 0.125% of collagenase for 1 h at 37 °C and an equal volume of 0.25% of trypsin-ethylenediaminetetra-acetic acid was added. The nerve segments were incubated for another 15 min at 37 °C followed by mechanical dissociation until they formed a homogeneous suspension. The cell pellets were obtained after centrifuging at 1000 rpm for 5 min at 4 °C. The supernatant was discarded, and the cells were then re-suspended in the complete culture medium and seeded on 35 mm tissue culture plates for further expansion. The purity of the primary cultured Schwann cells was confirmed by immunofluorescence staining of S100 proteins and the purity was at least 95% (Supplementary information).

### MTT assay

Scaffolds were sterilized through immersion in 70% (v/v) ethanol for 30 min at room temperature, subsequently they were exposed to ultraviolet light for 30 min. The Schwann cells viability on the scaffolds was quantified using MTT assay (*n* = 6/group, technical replication). Schwann cells were seeded on the scaffolds (*n* = 8 × 10^3^ per sample) and after 2 days, cell culture medium was replaced by 100 µl of MTT solution (0.5 mg/ml in PBS) and the plates were incubated at 37 °C for 3 h. Then, MTT solution was carefully replaced by 100 µl DMSO and the plates were gently agitated until the formed dark blue formazan crystals were dissolved and finally the absorbance of solution was read at 570 nm.

### Statistical analysis

Statistical analysis was performed using one-way analysis of variance using SPSS 20 software and difference was considered significant at *p* ≤ 0.05. All quantitative data were expressed as mean ± standard deviation.

## Results

### Morphological and chemical evaluation of PCL/Chit scaffolds before and after PEO fibers removal

The extent of sacrificial PEO fiber was tuned to 0, 20, 40, and 60% of the initial scaffold weight. To ensure that the designed combination of different syringes which were used for electrospinning resulted in the desired percentage of PEO within the PCL/Chit scaffolds, the actual percentages were verified through measuring the scaffolds weight before neutralization and also after PEO removal, since some PEO fibers may be removed during the neutralization step. The results are summarized in Table 2.

**Table 2:** The actual percentage of sacrificial PEO within the electrospun nanofibrous scaffolds was verified through measuring the scaffolds weight before and after PEO removal

Sample name	Estimated PEO percentage within the scaffolds	Scaffold weight before PEO removal (mg)	Scaffold weight after PEO removal (mg)	Calculated PEO percentage within the scaffolds
S-20% PEO	20%	6.4 ± 0.2	5.03 ± 0.15	21.67 ± 0.58
S-40% PEO	40%	4.93 ± 0.15	2.93 ± 0.15	40.67 ± 1.53
S-60% PEO	60%	5.97 ± 0.12	2.27 ± 0.15	61.33 ± 2.52

**Fig. 2:**
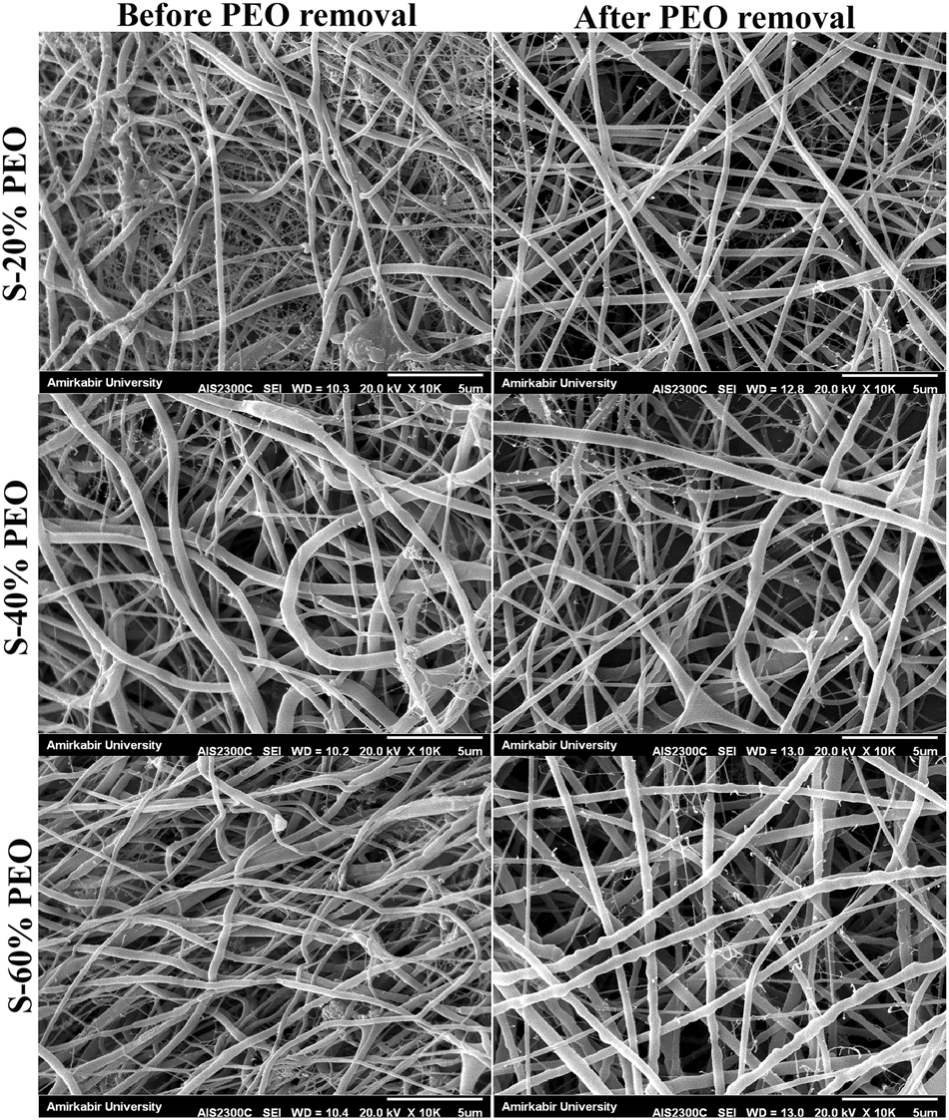
FE-SEM images of the PCL/Chit scaffolds with different percentages of PEO: 20% (S-20% PEO), 40% (S-40% PEO), and 60% (S-60% PEO) before and after removing of PEO

**Fig. 3:**
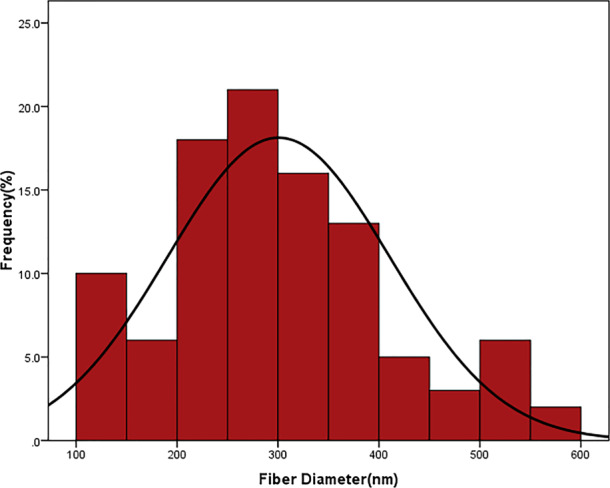
Fiber diameter distribution of PCL/Chit scaffold with 0% PEO

The microstructure and porosity of the scaffolds before and after removal of PEO fibers were verified by using FE-SEM. As it is shown in Fig. 2, a beadless nanofibrous structure was obtained and morphology of the fibers was well preserved after PEO removal which is clearly visible through the increase in interconnected pores within the scaffolds. The fiber diameter distribution of 0% PEO scaffold with average diameter of 300 ± 110 nm is also depicted in Fig. 3.

**Fig. 4:**
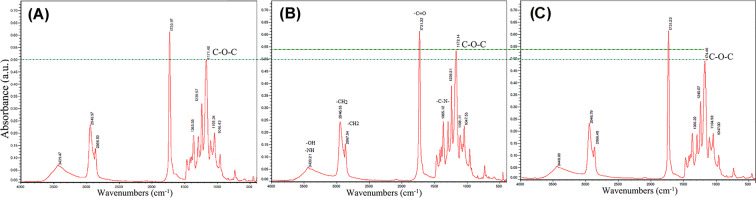
FTIR spectra of PCL/Chit scaffolds containing 0% PEO (**A**), 20% PEO before (**B**), and after (**C**) removal of PEO

FTIR spectra of PCL/Chit scaffolds containing 0% PEO and also 20% PEO before and after PEO removal are shown in Fig. 4. The peaks in the range of 1040–1180 cm^−1^ can be attributed to the C–O–C bond in PEO, PCL and chitosan, which were significantly reduced after PEO removal and reached its value in PCL/Chit scaffold containing 0% PEO.

### Porosity quantification

The porosity and pore size of the scaffolds with different percentages of PEO were calculated and summarized in Table 3. The scaffold with 60% PEO had more porosity and larger pore size than other samples. The difference between the values obtained from the two methods can be attributed to the measurement errors in the ethanol penetration method, since ethanol has a high evaporation rate. The porosities obtained from liquid displacement method were statistically significant between scaffolds prepared using different concentrations of PEO (i.e., 0–60%) (*p* ≤ 0.05). However, for apparent porosity and pore size the significant difference was observed between the scaffolds prepared using 20, 40 and 60% PEO (*p* ≤ 0.05) and there was no statistically significant difference between 0 and 20% PEO.

**Table 3:** Porosity and pore size of the scaffolds with different percentages of PEO removal which were determined using two methods: Liquid displacement method and Apparent porosity (*p ≤ 0.05 for scaffolds prepared using different concentration of PEO)

PEO %	Porosity measured by liquid displacement (%)	Apparent porosity (%)	Pore size (µm)
0	58.3 ± 2.08*	53 ± 3.61	3.52 ± 0.078
20	63.67 ± 0.58*	57.67 ± 1.53*	3.65 ± 0.033*
40	79.67 ± 0.58*	75.33 ± 1.16*	4.46 ± 0.069*
60	91 ± 1*	81 ± 1*	4.9 ± 0.101*

### WCA measurement

**Fig. 5:**
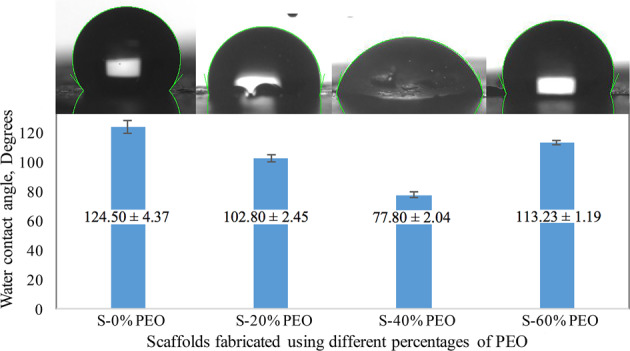
Water contact angle (WCA) values for PCL/Chit scaffolds with different percentages of PEO: 20% (S-20% PEO), 40% (S-40% PEO), and 60% (S-60% PEO). The experiment was conducted after PEO fiber removal

Figure 5 demonstrates the WCA values for scaffolds with different percentages of PEO and the experiment was conducted after PEO fibers removal. The WCA value on the PCL/Chit scaffold was 124.50° ± 4.37. Different weight percentages of PEO, as sacrificial fiber, were incorporated within the PCL/Chit scaffold during electrospinning process in order to improve the scaffold porosity. Using 20% and 40% wt of PEO led to an increase in wettability of the scaffolds, the measured WCA was 102.80° ± 2.45 and 77.80° ± 2.04, respectively. However, further increase in the extent of porosity to 60% wt decreased the scaffolds wettability to 113.23° ± 1.19.

### Physicochemical properties of AuNP-Decorated PCL/Chit nanofibrous scaffolds

**Fig. 6:**
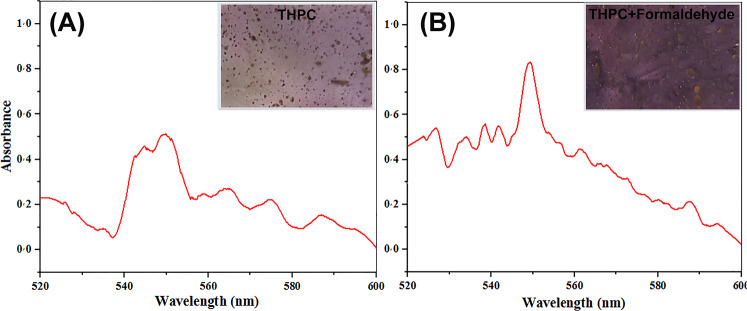
UV-Visible absorbance spectra of the conductive scaffolds fabricated using THPC alone (**A**) and in combination with formaldehyde (**B**). The inset shows optical images of conductive scaffolds fabricated using THPC alone or in combination with formaldehyde

Based on the results WAC, the sample with 40% PEO (75–80% porosity after PEO removal) was selected to perform further evaluations. In situ synthesis of AuNPs on the PCL/Chit nanofibers was revealed by immediate color change of the PCL/Chit scaffold after immersion in basic solution of THPC. Further reduction of AuCl_4_^−^ using formaldehyde solution resulted in the color change to a dark purple-brownish. These changes in color indicated the formation of AuNPs on the scaffold as shown in Fig. 6.

To further verify the presence of AuNPs on the scaffolds, UV–Vis spectroscopy was conducted and the absorbance spectra for AuNP-decorated scaffolds which were fabricated by using either THPC alone or in combination with formaldehyde are shown in Fig. 6. The resonance peak around 550 nm which is observable in spectra of both samples is near the plasmon resonance of AuNPs in a solution (i.e., 520 nm) confirming the formation of AuNPs on both scaffolds.

**Fig. 7:**
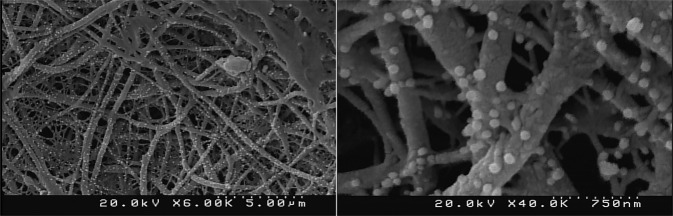
FE-SEM images of the AuNPs-decorated PCL/chitosan scaffolds which were fabricated by using both reducing agents of THPC and formaldehyde, at two different magnification

Because of higher amount of electrical conductivity; AuNP-decorated scaffolds which were fabricated by using both reducing agents of THPC and formaldehyde were chosen for further evaluations and FE-SEM images of these scaffolds are shown in Fig. 7. As shown, the AuNPs were deposited homogeneously on entire nanofibers and the fibrous structure of the scaffold was well preserved during the gold reduction process. Furthermore, the synthesized AuNPs exhibited a spherical shape and a narrow particle size distribution with a mean particle diameter of 126 ± 20 nm. Also, quantification of Au content in the scaffold using energy-dispersive X-ray showed that there was 10.4 ± 1.13 wt% of gold within the scaffold fabricated using both reducing agents of THPC and formaldehyde. The elemental composition of AuNP-decorated scaffold and its corresponding spectra and surface mapping are reported in supplementary information.

### Electrical conductivity of AuNP-decorated PCL/Chit nanofibrous scaffolds

By using the two-point probe method, electrical flow-voltage curves were obtained for AuNPs-decorated scaffolds. The electrical conductivity (*σ*) of these scaffolds was obtained through the following equations (Eqs. (7) and (8)), where R is the slope of the current flow-voltage curve and *ρ*, *L*, *W* and *t* are resistivity, length, width and thickness of the scaffold, respectively. The electrical conductivity of AuNP-decorated scaffolds which were fabricated by using THPC alone or in combination with formaldehyde are reported in Table 4.

7 $${\mathrm{R = }}\,\rho {\mathrm{L/Wt}}$$

8 $${\upsigma} = 1/{\uprho}$$

**Table 4:** Electrical conductivity of the AuNP-decorated scaffolds fabricated through in situ gold ion reduction using either THPC alone or in combination with formaldehyde (*p* ≤ 0.05)

The used reducing agent(s)	Electrical Conductivity (S m^−1^)
THPC	3.125 ± 0.75
THPC + Formaldehyde	4.44 ± 0.83

### Schwann cell behavior on the AuNP-decorated scaffolds

**Fig. 8:**
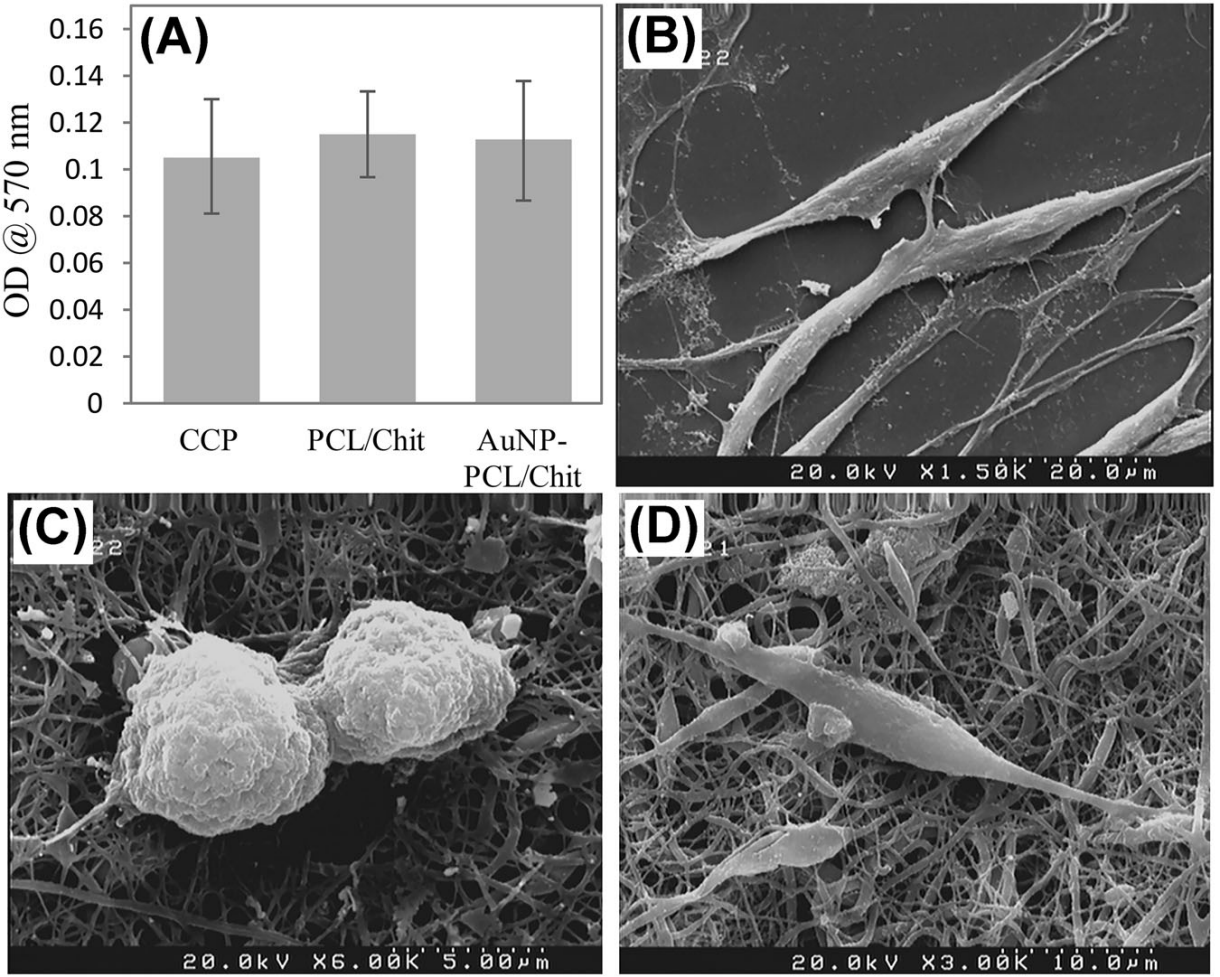
**A** MTT assay showing Schwann cell proliferation on cell culture plate (CCP), PCL/chitosan (PCL/Chit) and AuNPs-decorated PCL/Chit scaffolds (AuNP-PCL/Chit) at 48 h after cell seeding. **B** FE-SEM microgrphs of Schwann cells on tissue culture plate (**A**) and AuNPs-decorated PCL/Chit scaffolds which were fabricated using THPC in combination with formaldehyde (**B** and **C**). The characteristic morphology of mature Schwann cells in vitro, spindle-shape with long processes, was evident on both tissue culture plate and AuNPs-decorated PCL/Chit scaffolds. Also, there was some proliferating Schwann cells on AuNPs-decorated PCL/Chit scaffolds (**B**)

The viability of Schwann cells on PCL/chit scaffolds with 40% PEO (with 75–80% porosity after PEO removal) with and without AuNPs was evaluated by MTT assay at 48 h after cell seeding. As shown in Fig. 8A, gold decoration of PCL/Chit nanofibers had no cytotoxic effect.

Also, the FE-SEM micrographs of Schwann cells on the cell culture plate and AuNPs-decorated scaffold are shown in Fig. 8B–D, respectively. Similar to the cell culture plate, as the positive control, morphology of Schwann cells was spindle-shape with long processes, the characteristic morphology of mature Schwann cells in vitro. Also, there were some proliferating Schwann cells on the AuNP-decorated scaffold (Fig. 8C).

## Discussion

This study represents a novel method for fabrication of conductive microporous scaffolds through in situ reduction of gold ions on the electrospun PCL/Chit nanofibers. Tissue engineered scaffolds should be biocompatible, biodegradable and non-immunogenic. Apart from these characteristics, nanofibrous microstructure with interconnected pores as well as electrical conductivity can prominently influence nerve regeneration [24]. Cryogenic electrospinning [25], gas foaming [26] and salt leaching [27] are among the methods used for production of porous scaffolds which form either large or isolated pores. On the other hand, sacrificial fiber method produces interconnected fibrous pores and also provides a good control over the pore size [19].

Fibrous structures helps neural cells align in one direction which is closer to the structure of the neurons [28–30]. Although aligned fibers can improve Schwann cell alignment [31], it also reduces the scaffold’s strength [32]. Since the method used for in situ synthesized AuNPs in our study weakened the mechanical strength of the PCL/Chit scaffolds, we selected a much stronger nanofibrous scaffold with random orientation to study the scaffold’s conductivity. Nanofibrous scaffolds with different porosity percentages were fabricated using PEO as sacrificial material along with electrospinning, and the optimum porosity percentage was selected according to the hydrophilicity of the scaffolds (with 40% PEO and porosity of 75–80%). The hydrophilic/hydrophobic properties of the biomaterials influence the cell adhesion and consequently cell viability, and proliferation [33]. Generally, it has been found that cell attachment improves when surface hydrophobicity of the material increases [34]. Specifically, it was shown that surface wettability can potentially influence neurite formation and elongation and a water contact angle around 55 degree has been introduced as a suitable hydrophilic properties for axon regeneration [35]. Also, It is reported for Schwann cells that maximum cell density is obtained not for the most hydrophobic material, but for the copolymer having a 10 wt% of the hydrophilic component [36].

Surface roughness has a strong effect on the contact angle and wettability of a surface. The effect of roughness depends on whether the droplet wet the surface grooves or air pockets are left between the droplet and the surface. If the surface is wetted homogeneously, the droplet is in Wenzel state. In Wenzel state, an increase in the surface roughness enhances the wettability due to the chemistry of the surface. If the surface is wetted heterogeneously, the droplet is in Cassie–Baxter state. In this state elevation of the surface roughness leads to an increase in the hydrophobicity of the surface [37]. Figure 5 does not show a regular pattern of water contact angle changes which can be due to the competition of two above-mentioned principles. Based on the surface chemistry, the PCL/chit electrospun scaffolds are hydrophilic and the droplet must be in Wenzel state, however in the sample with 60% porosity the amount of air pocket overcomes the surface chemistry which shows Cassie-Baxter state of the droplet. In conclusion, 75–80% porosity with WCA of 77.80° ± 2.04 is a ballance of these two states, therefor the PCL/Chit scaffolds prepared using 40% PEO was selected as an appropriate substrate for further evaluations. It is also reported that when the ratio of PCL/Chit is 2.5, the porous sample with 35 μm pore size is definitely hydrophilic with a water contact angle of 70.92° ± 2.99 [38].

In addition to topographical signals, electrical signal and conductive scaffolds have attracted a great attention for regeneration of various tissues [39, 40]. Polyaniline and polypyrrole are in the group of conductive polymers; however, their non-degradability has limited their application in tissue engineering. Some studies have reported the use of carbon nanotubes to fabricate a conductive scaffold, but the cytotoxicity of this material is highly debatable. The use of piezoelectric polymers is also not practical owing to difficulty in fabrication [2]. Therefore, using AuNPs for fabrication of electrically conductive scaffolds could be a rational choice due to its biocompatibility, easy construction, nano dimensions with high surface-to-volume ratio and ability of binding to chemical agents [41].

The critical element of neuronal communication is the action potential made at the synapse, therefore, a conductive scaffold can have potential effect on cell communication and enhance nerve regeneration. In order to have a suitable electrical simulation, a physiological electrical potential should be within an order of millivolts (mV). For example, 100 mV is a suitable amount [42]. Furthermore, an electrical current in the range of 0.6–400 μA has been demonstrated to be biologically effective in both in-vitro and in-vivo studies [2]. Hence, scaffolds with conductivity higher than 0.1 S m^−1^ without the signs of cellular toxicity can be considered as a proper candidate for tissue engineering application [13, 43].

In a study, polypyrrole (PPy) nanotube was used as both supporting template and reducing agent for in situ reduction of HAuCl_4_. They achieved AuNPs with diameter of 13 nm; however, the conductivity of the PPy/Au nanocomposites were much lower than that of the PPy nanotubes because of over‐oxidization of PPy. In addition, the Au nanoparticles did not form a conducting network due to their low population density on the PPy nanotubes [44]. In a recent study, gold nanoparticles of about 50 nm were fabricated and used to decorate the aligned polyurethane (PU) nanofibers in order to increase the neurite elongation. Generally, PU nanofiber is highly hydrophobic, however its WCA decreased after functionalization with poly(lactide acid) and decoration with gold nanoparticles from 155.9° to 107°. Gold nanoparticles were able to better transfer the current along the cell under electrical stimulation inside the bioreactor system, and increased the neurite outgrowth and elongation [45]. However, one should consider the very low degradation rate of PU as a biomaterial for nerve tissue engineering.

The composite of AuNPs can also be obtained by simultaneous electrospinning of AuNPs suspension with polymer solutions, which need a stable suspension of particles acquired by AuNPs modification [46, 47]. This limitation has led many researchers to use the approach of in situ reducing AuNPs instead of simultaneous electrospinning of AuNPs with polymer solution. Chitosan can act as a reducing agent for AuNPs through the conversion of its hydroxyl groups to carboxyl groups [48]. In our previous study the reducing property of chitosan was utilized for the reduction of AuNPs on PCL/Chit scaffold resulting in the scaffold conductivity of 1.16 S m^−1^ [14]. In order to improve the conductivity, two reducing agents of THPC and formaldehyde were selected in this study. The mechanism of these reducing agents in aqueous solutions has been widely studied [49, 50]. The use of these agents resulted in faster reduction of gold ions, higher number of absorbed AuNPs and therefore significantly higher conductivity up to 4.44 S m^−1^ compared to using of chitosan alone. In addition, a decrease in nanoparticle diameter from 175 ± 69 nm to 126 ± 20 nm was observed in comparison to our previous study. The scaffolds reduced using both THPC and formaldehyde showed a better conductivity compared to the scaffolds reduced with THPC alone. Moreover, the results proved that interconnected porosity of the scaffolds which was increased using sacrificial fiber method and the morphological structure of nanofibrous scaffold were well preserved upon removal of the sacrificial fibers. As WCA measurements showed, these porous nanofibrous scaffolds are hydrophilic and suitable for cell attachment and spreading. Finally, FE-SEM results confirmed cells adhesion and proliferation after seeding on the conductive nanofibrous scaffolds.

## Conclusions

In this study, the interconnected porosity of the PCL/chitosan scaffolds was improved by using sacrificial fiber method resulting in higher surface hydrophilicity. Among the scaffolds with different porosity, the sample with 40% PEO, with 75–80% porosity after PEO removal, showed the highest hydrophilicity. The reducing agents of THPC and formaldehyde were also successfully used to increase the reduction rate of gold nanoparticles and conductivity of scaffolds. In addition, Schwann cell viability test as well as morphological evaluations demonstrated that enhancing the porosity of the electrospun nanofibrous scaffolds can improve scaffold hydrophilicity and consequently cell adhesion, spreading and prolifratation.

## Supplementary information

Supplementary information
