# Supplementary material for: Fabrication and evaluation of porous and conductive nanofibrous scaffolds for nerve tissue engineering
Source: J Mater Sci Mater Med. 2021 Apr 13;32(4):46. doi: 10.1007/s10856-021-06519-5 (PMC8043924; doi:10.1007/s10856-021-06519-5)
Supplement: Supplementary file 1 — Supplementary information [file 10856_2021_6519_MOESM1_ESM.pdf]

Supporting information

Fabrication and evaluation of the porous and conductive nanofibrous scaffolds for nerve tissue engineering

Yasaman Pooshidani[a]†, Nastaran Zoghi [b]†, Mina Rajabi[c], Masoumeh Haghbin Nazarpak[d], Zahra Hassannejad [e*]

[a] Departmant of Biomedical Engineering, Amirkabir University of Technology (Tehran Polytechnic), Tehran, Iran

[b] Department of Biochemistry, Faculty of Biological Sciences, Tarbiat Modares University, Tehran, Iran

[c] Centre for Bioengineering and Nanomedicine, University of Otago, Dunedin, New Zealand

[d] New Technologies Research Center (NTRC), Amirkabir University of Technology (Tehran Polytechnic), Tehran, Iran

[e] Pediatric Urology and Regenerative Medicine Research Center, Children's Medical Center, Tehran University of Medical Sciences, Tehran, Iran

†Authors with the same contribution to this paper

*** To whom correspondence may be addressed:**

Zahra Hassannejad,

Email: zhassannejad@sina.tums.ac.ir

Phone: +98 21 66565400

Fax: +98 21 66565500

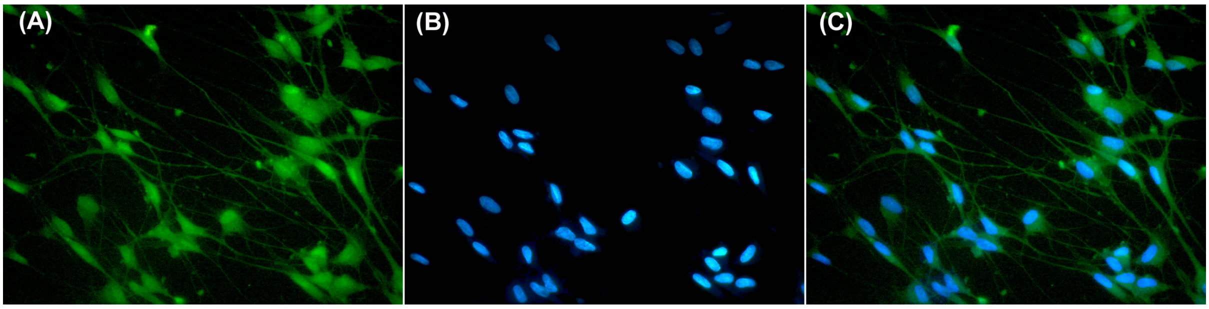

Figure S1: The purity of the primary cultured Schwann cells was confirmed by immunofluorescence staining of S100 proteins. To quantify Schwann cell purity, the number of positive cells for S100 staining (green) was counted and divided by the number of cell nuclei (blue). The purity of Schwann cells was more than 95%. (A) immunofluorescence staining of primary cultured Schwann cells against S100 (green), as the specific biomarker of Schwann cells, (B) the corresponding nuclei stained with Hoechst (blue) and (C) image merged from (A) and (B).

(A) Selected areas of the scaffold	Weight%			
	C	O	N	Au
Area 1	57.9	30.9	1.6	9.6
Area 2	61.6	27.7	0.0	11.2
Average ± SD	59.75 ± 1.85	29.3 ± 1.6	0.8 ± 0.8	10.4 ± 0.8

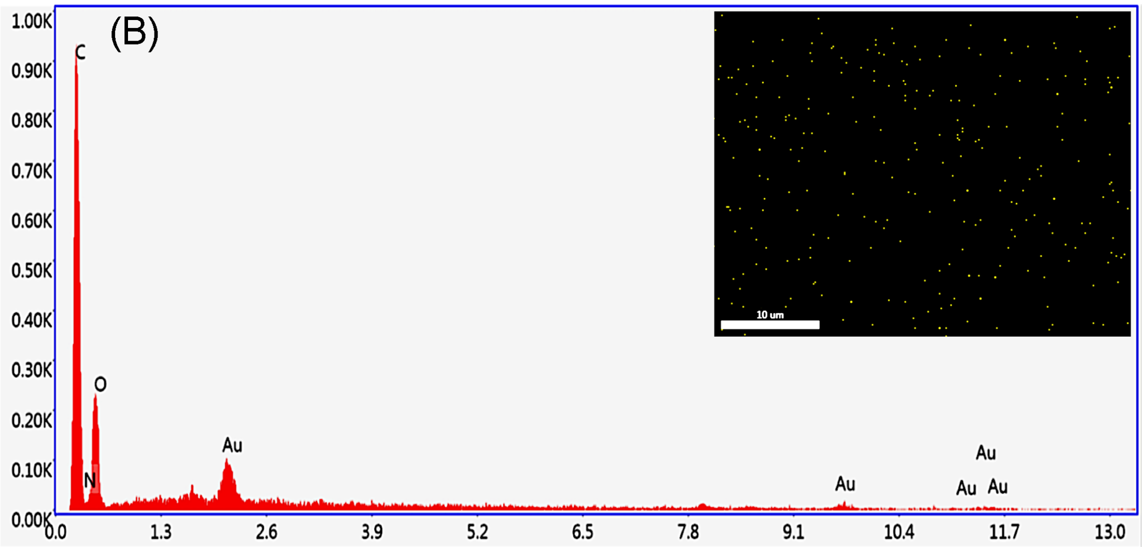

Figure S2: The elemental composition of AuNP-decorated scaffold which was fabricated using both reducing agents of THPC and formaldehyde, from two different areas of the scaffold (A) and its corresponding EDX spectrum and surface mapping (B).
